# Supplementary material for: Genome-Wide Identification of Bilberry WRKY Transcription Factors: Go Wild and Duplicate
Source: Plants (Basel). 2023 Sep 5;12(18):3176. doi: 10.3390/plants12183176 (PMC10535657; doi:10.3390/plants12183176)
Supplement: Supplementary file 1 [file Figure S5.pdf › Figure S5.pdf]

Genome-Wide Expression of WRKY Transcription Factors in Blueberry: Go Wild and Duplicate

Winder Felipez [1,2,†], Jennifer Villavicencio [2,3,†], Valeria Oliveira Nizolli [2], Camila Pegoraro [2], Luciano da Maia [2] and Antonio Costa de Oliveira [2,*]

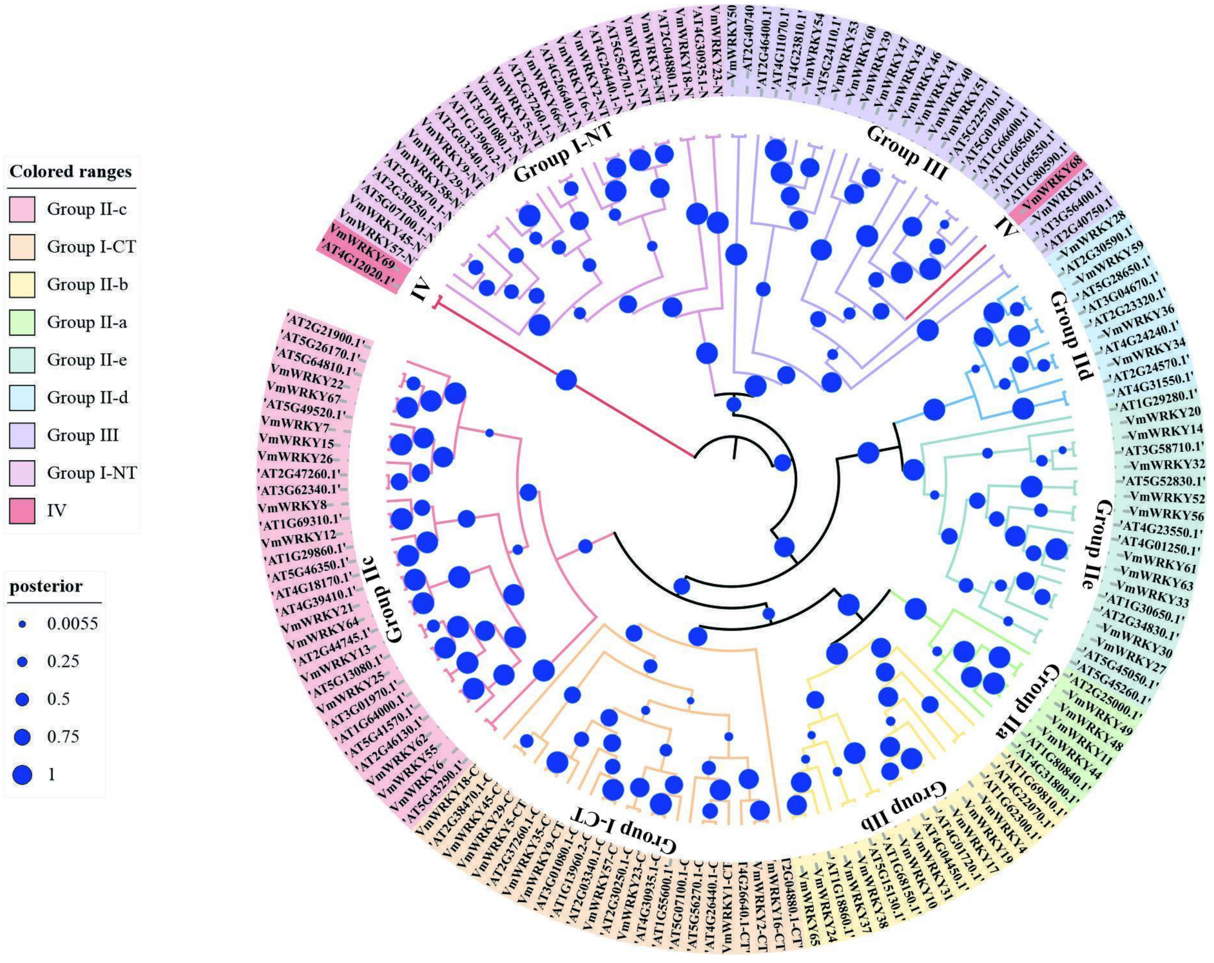

Figure S5: The unrooted phylogenetic tree was generated with the BEAST v.2.5 software, using the complete amino acid sequences of the 72 WRKY proteins from *Arabidopsis thaliana* genome and 69 WRKY proteins from *Vaccinium myrtillus* genome by Neighbor-Joining method, with the UPGMA clustering method and 1000 bootstrap repeats. WRKY protein subfamilies (I, IIa, IIb, IIc, IId, IIe and III).
