# Supplementary material for: Genome-Wide Identification of Bilberry WRKY Transcription Factors: Go Wild and Duplicate
Source: Plants (Basel). 2023 Sep 5;12(18):3176. doi: 10.3390/plants12183176 (PMC10535657; doi:10.3390/plants12183176)
Supplement: Supplementary file 1 [file plants-12-03176-s001.zip › Figure S10.pdf]

Genome-Wide Expression of WRKY Transcription Factors in Blueberry: Go Wild and Duplicate

Winder Felipez [1,2,†], Jennifer Villavicencio [2,3,†], Valeria Oliveira Nizolli [2], Camila Pegoraro [2], Luciano da Maia [2] and Antonio Costa de Oliveira [2,*]

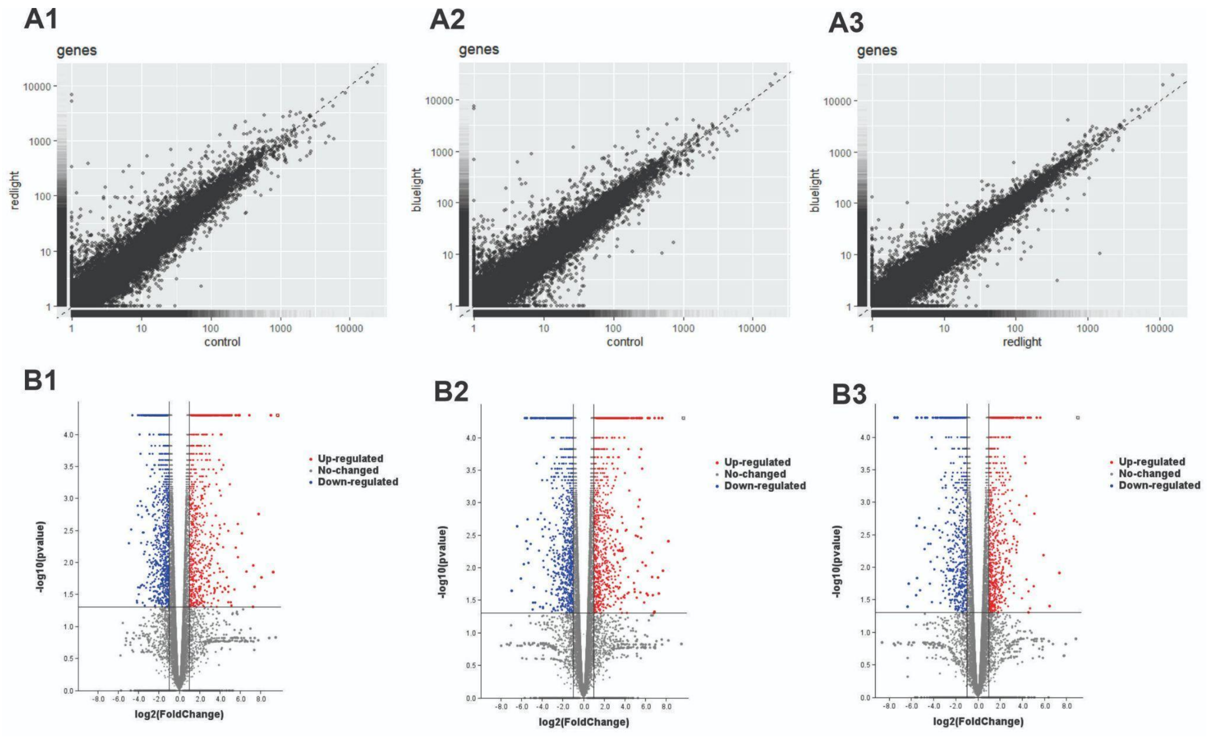

Figure S10: Analysis of gene expression in samples of *Vaccinium myrtillus*: A1 - A3) Volcano plots reveal genes that differ significantly between the pairs of conditions control and red light, control and blue light, red light and blue light. B1 - B3) CummeRbund scatter plots highlight general similarities and specific outliers between conditions control and red light, control and blue light, red light and blue light.
