# Supplementary material for: Genome-Wide Identification of Bilberry WRKY Transcription Factors: Go Wild and Duplicate
Source: Plants (Basel). 2023 Sep 5;12(18):3176. doi: 10.3390/plants12183176 (PMC10535657; doi:10.3390/plants12183176)

Genome-Wide Expression of WRKY Transcription Factors in Blueberry: Go Wild and Duplicate

Winder Felipez [1,2,†], Jennifer Villavicencio [2,3,†], Valeria Oliveira Nizolli [2], Camila Pegoraro [2], Luciano da Maia [2] and Antonio Costa de Oliveira [2,*]

Figure S2: Sequence Logo visualization of the identified conserved motives in the WRKY proteins sequence using Expectation Maximization Tool for Motif Elicitation MEME.

Motif 1

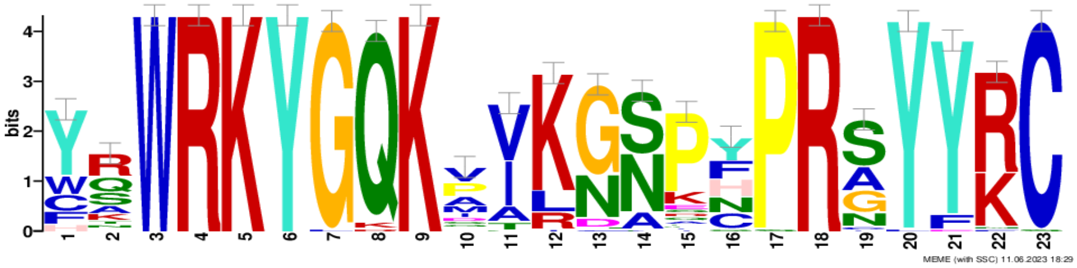

Motif 2

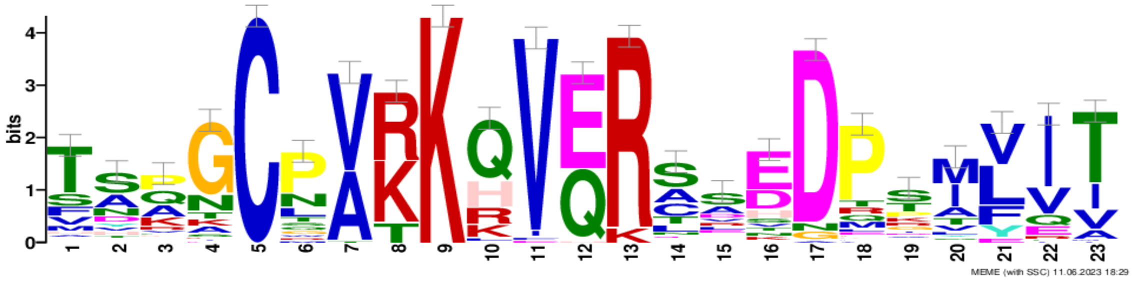

Motif 3

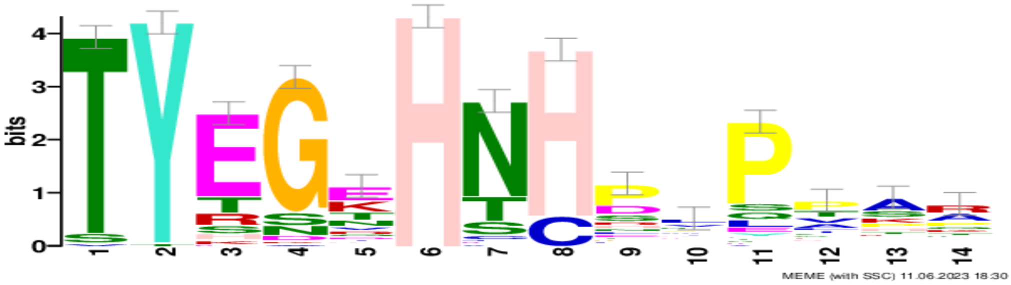

Motif 4

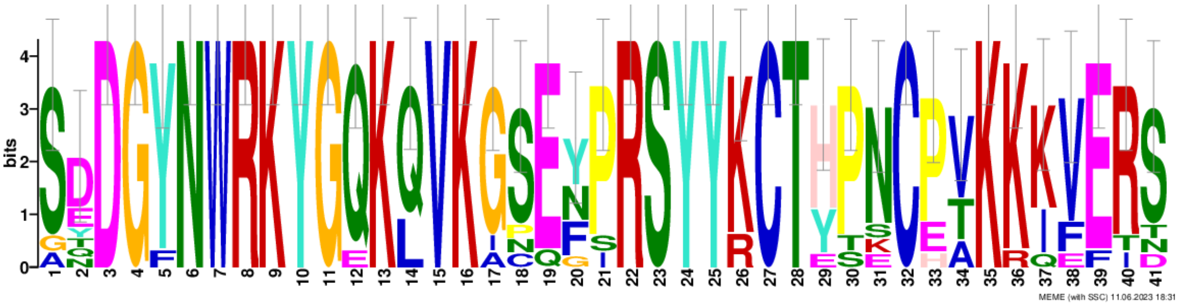

Motif 5

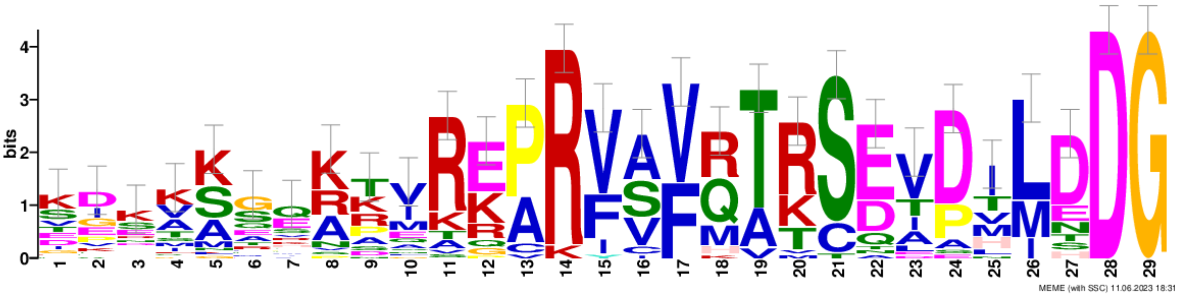

Motif 6

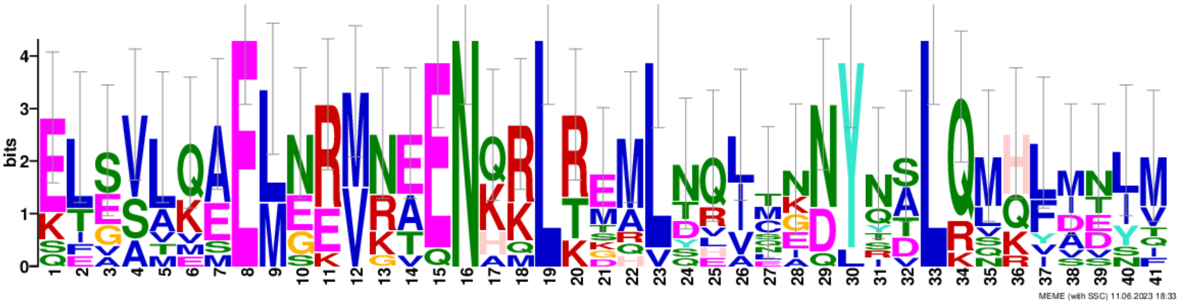

Motif 7

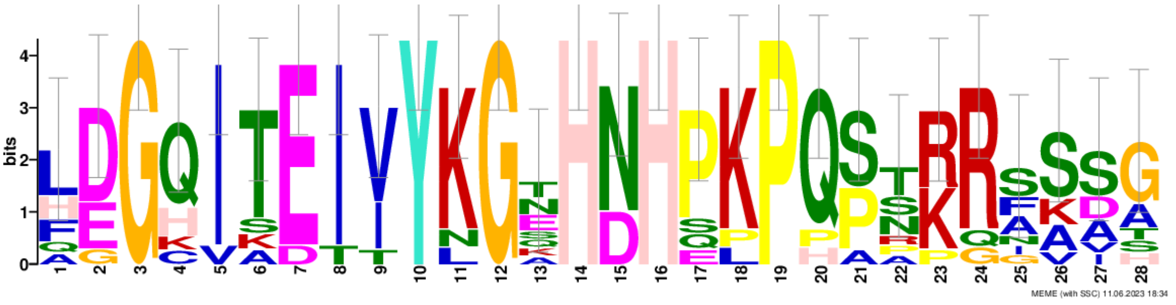

Motif 8

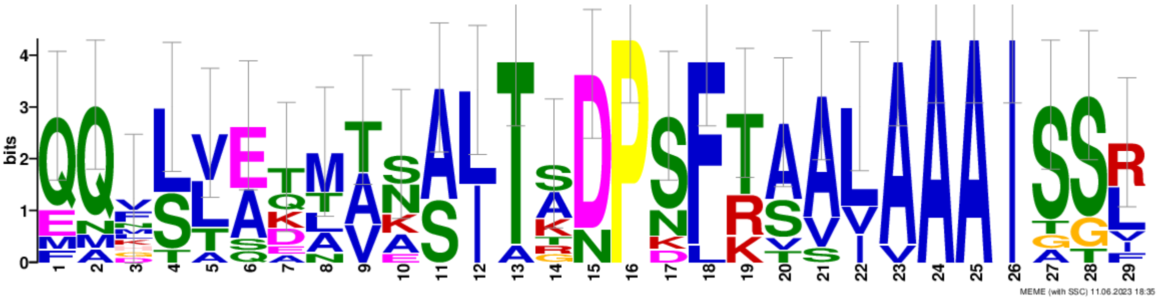

Motif 9

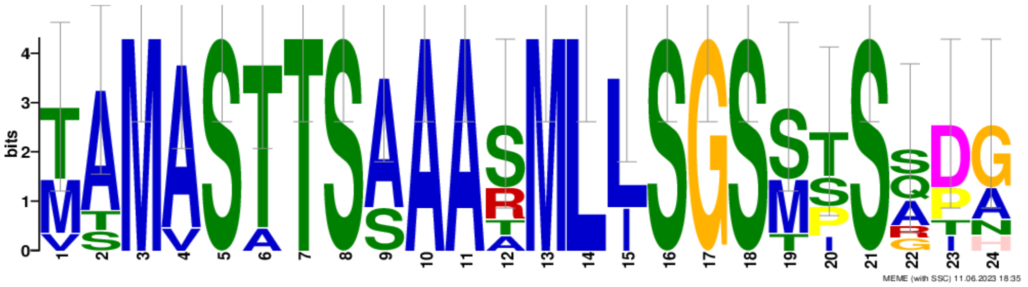

Motif 10

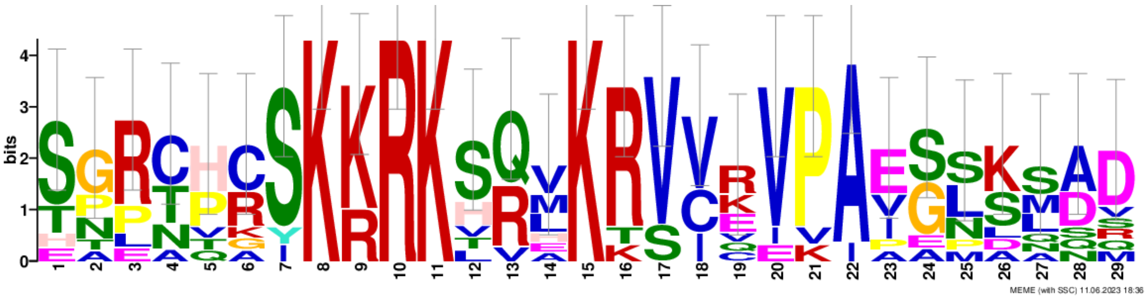

Motif 11

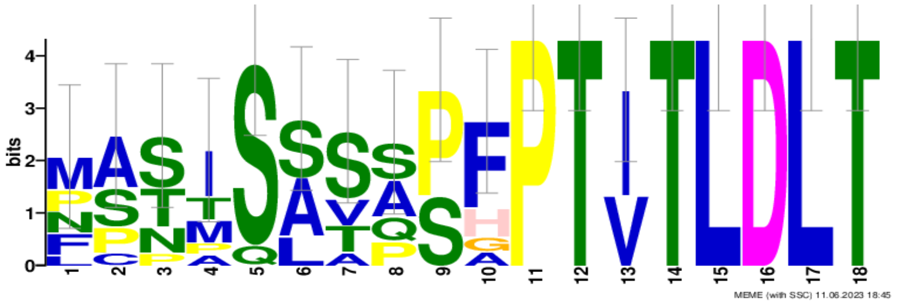

Motif 12

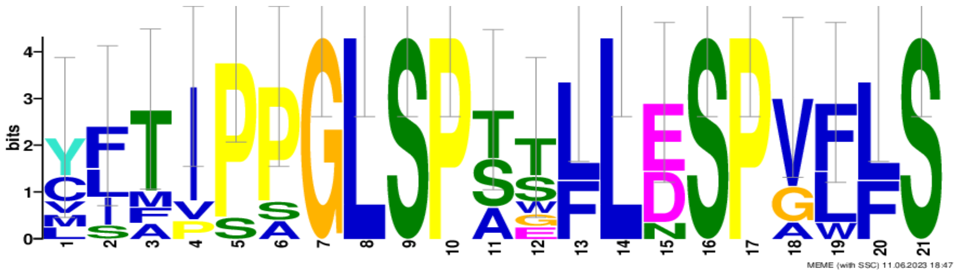

Motif 13

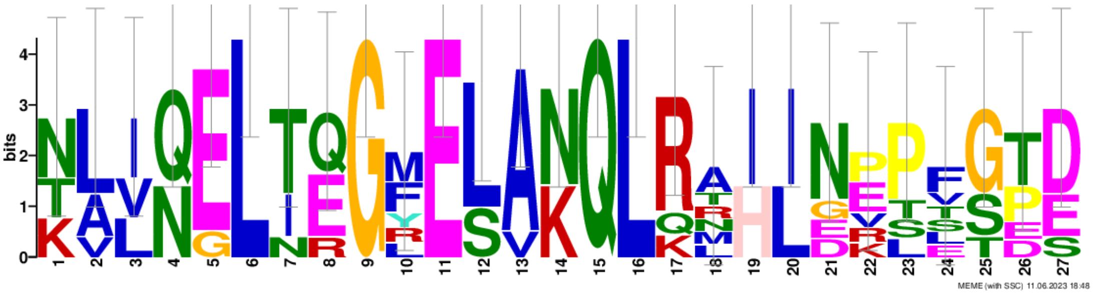

Motif 14

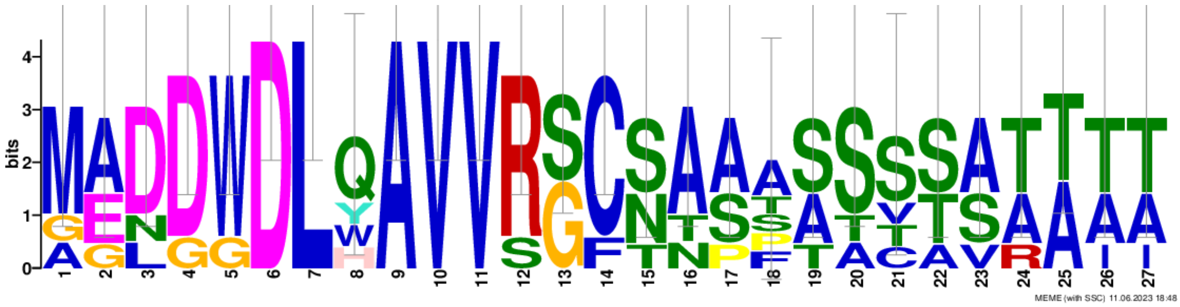

Motif 15

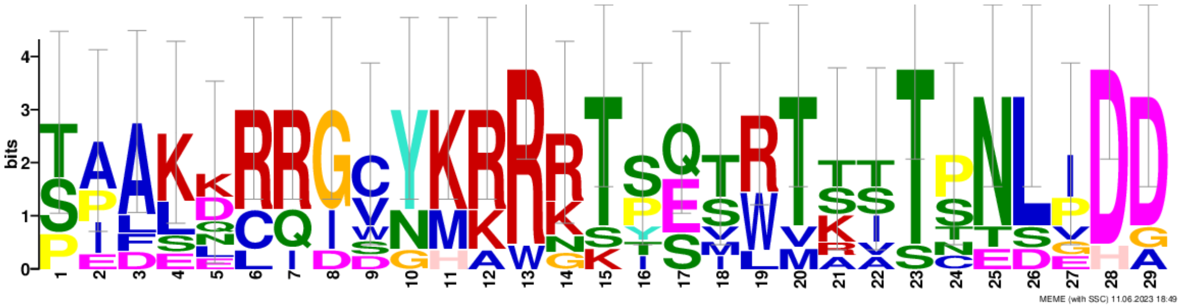

Motif 16

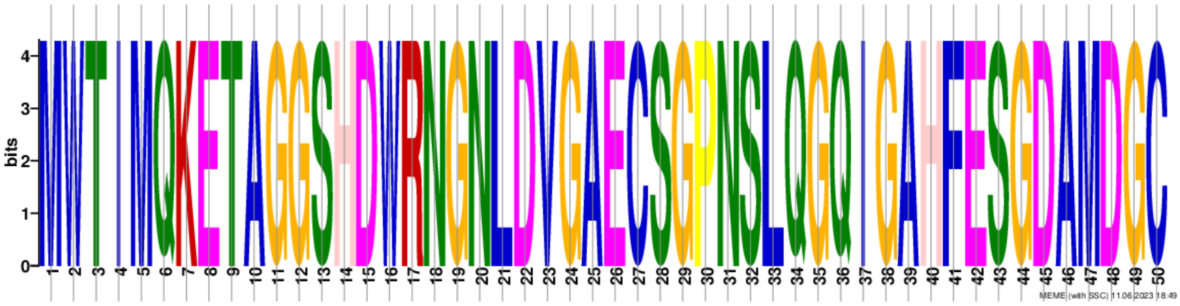

Motif 17

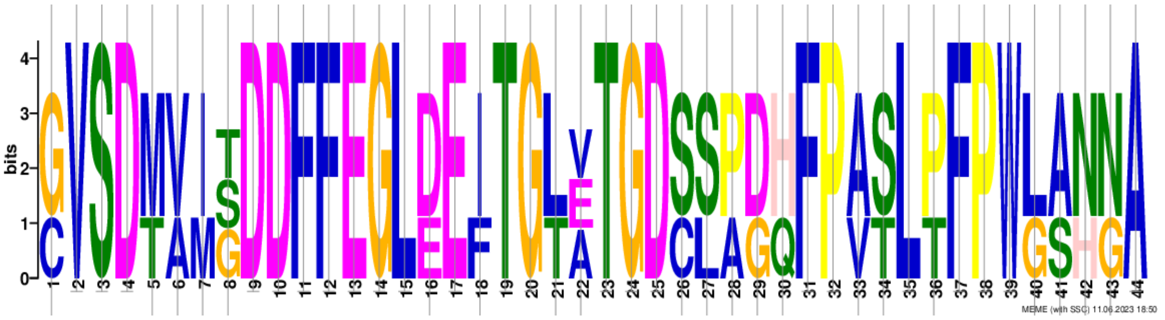

Motif 18

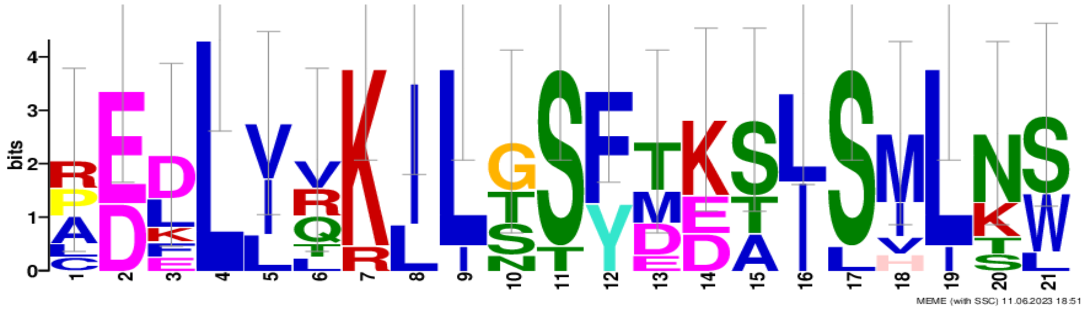

Motif 19

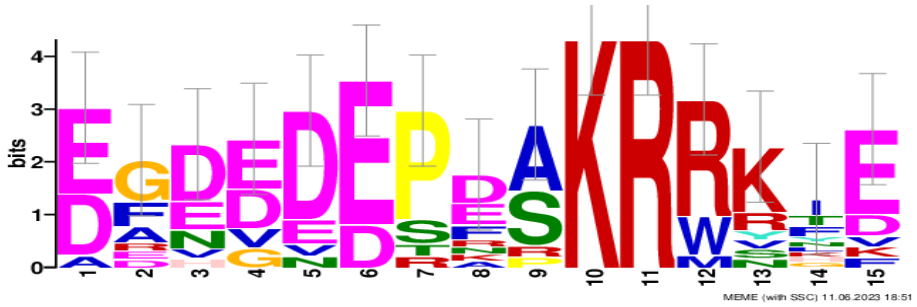

Motif 20

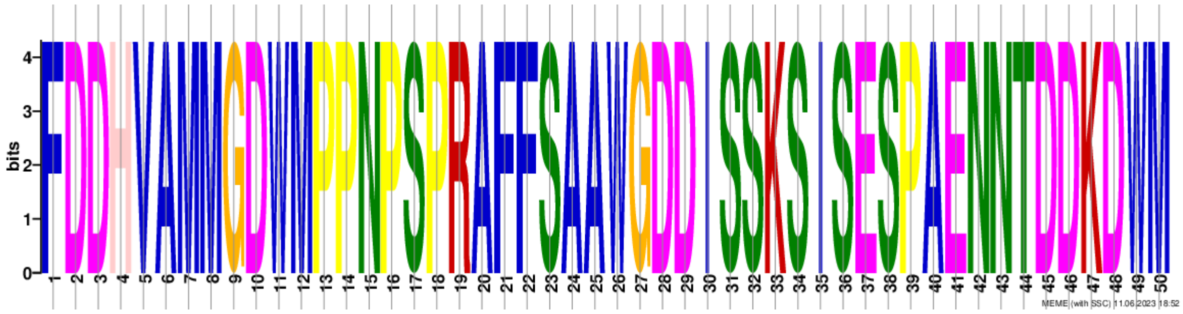

Supplement: Supplementary file 1 [file plants-12-03176-s001.zip › Figure S2.pdf]
