# Supplementary material for: Genome-Wide Identification of Bilberry WRKY Transcription Factors: Go Wild and Duplicate
Source: Plants (Basel). 2023 Sep 5;12(18):3176. doi: 10.3390/plants12183176 (PMC10535657; doi:10.3390/plants12183176)
Supplement: Supplementary file 1 [file plants-12-03176-s001.zip › Figure S3.pdf]

Genome-Wide Expression of WRKY Transcription Factors in Blueberry: Go Wild and Duplicate

Winder Felipez [1,2,†], Jennifer Villavicencio [2,3,†], Valeria Oliveira Nizolli [2], Camila Pegoraro [2], Luciano da Maia [2] and Antonio Costa de Oliveira [2,*]

Paralogous Pairs	Identity	Similarity (>70%)	Gaps	Score	Distance (in Chromosome < 100kb)	Tandem duplicates
VmWRKY2–VmWRKY3	134/399 (33.6%)	178/399 (44.6%)	114/399 (28.6%)	458	4,421	N
VmWRKY26–VmWRKY27	58/190 (30.5%)	95/190 (50.0%)	13/190 (6.8%)	228,5	102,076	N
**VmWRKY37–VmWRKY38**	**349/417 (83.7%)**	**368/417 (88.2%)**	**4/417 (1.0%)**	**1770**	**23,629**	**Y**
**VmWRKY40–VmWRKY41**	**240/241 (99.6%)**	**240/241 (99.6%)**	**0/241 (0.0%)**	**1276**	**37,603**	**Y**
VmWRKY40–VmWRKY42	43/86 (50.0%)	58/86 (67.4%)	5/86 (5.8%)	235,5	64,764	N
VmWRKY40–VmWRKY43	34/100 (34.0%)	46/100 (46.0%)	22/100 (22.0%)	152	68,637	N
VmWRKY41–VmWRKY42	43/86 (50.0%)	58/86 (67.4%)	5/86 (5.8%)	235,5	23,667	N
VmWRKY41–VmWRKY43	34/100 (34.0%)	46/100 (46.0%)	22/100 (22.0%)	152	27,54	N
VmWRKY42–VmWRKY43	44/87 (50.6%)	56/87 (64.4%)	12/87 (13.8%)	230,5	3,357	N
**VmWRKY46–VmWRKY47**	**209/335 (62.4%)**	**247/335 (73.7%)**	**15/335 (4.5%)**	**1007**	**7,765**	**Y**
**VmWRKY48–VmWRKY49**	**129/184 (70.1%)**	**146/184 (79.3%)**	**11/184 (6.0%)**	**692**	**51,301**	**Y**
VmWRKY62–VmWRKY63	52/193 (26.9%)	69/193 (35.8%)	72/193 (37.3%)	188	57,14	N
VmWRKY55–VmWRKY56	47/141 (33.3%)	65/141 (46.1%)	27/141 (19.1%)	178,5	19,538	N
VmWRKY58–VmWRKY59	36/98 (36.7%)	51/98 (52.0%)	18/98 (18.4%)	156	4,016	N

Figure S3: Pairwise identities between paralogous pairs of *VmWRKY* genes - Y:yes, N: no.
