# Supplementary material for: Genome-Wide Identification of Bilberry WRKY Transcription Factors: Go Wild and Duplicate
Source: Plants (Basel). 2023 Sep 5;12(18):3176. doi: 10.3390/plants12183176 (PMC10535657; doi:10.3390/plants12183176)
Supplement: Supplementary file 1 [file plants-12-03176-s001.zip › Figure S4.pdf]

Genome-Wide Expression of WRKY Transcription Factors in Blueberry: Go Wild and Duplicate

Winder Felipez [1,2,†], Jennifer Villavicencio [2,3,†], Valeria Oliveira Nizolli [2], Camila Pegoraro [2], Luciano da Maia [2] and Antonio Costa de Oliveira [2,*]

Paralogous Pairs	Identity	Similarity (>70%)	Gaps	Score	Segmental duplicates
VmWRKY31–VmWRKY17	254/557 (45.6%)	328/557 (58.9%)	114/557 (20.5%)	1064	N
**VmWRKY4–VmWRKY19**	**347/568 (61.1%)**	**400/568 (70.4%)**	**89/568 (15.7%)**	**1556,5**	**Y**
**VmWRKY65–VmWRKY24**	**378/387 (97.7%)**	**379/387 (97.9%)**	**2/387 (0.5%)**	**1938**	**Y**
**VmWRKY11–VmWRKY44**	**226/344 (65.7%)**	**259/344 (75.3%)**	**25/344 (7.3%)**	**1050**	**Y**
VmWRKY56–VmWRKY30	114/340 (33.5%)	151/340 (44.4%)	115/340 (33.8%)	397	N
**VmWRKY14–VmWRKY32**	**172/288 (59.7%)**	**202/288 (70.1%)**	**37/288 (12.8%)**	**813**	**Y**
**VmWRKY61–VmWRKY63**	**289/321 (90.0%)**	**298/321 (92.8%)**	**7/321 (2.2%)**	**1517,5**	**Y**
VmWRKY36–VmWRKY34	192/350 (54.9%)	244/350 (69.7%)	31/350 (8.9%)	904,5	N
**VmWRKY59–VmWRKY28**	**258/349 (73.9%)**	**293/349 (84.0%)**	**16/349 (4.6%)**	**1326**	**Y**
VmWRKY15–VmWRKY7	204/331 (61.6%)	230/331 (69.5%)	60/331 (18.1%)	950	N
VmWRKY12–VmWRKY8	106/246 (43.1%)	134/246 (54.5%)	38/246 (15.4%)	437,5	N
**VmWRKY67–VmWRKY22**	**94/129 (72.9%)**	**101/129 (78.3%)**	**1/129 (0.8%)**	**489**	**Y**
VmWRKY21–VmWRKY64	104/234 (44.4%)	127/234 (54.3%)	34/234 (14.5%)	421	N
**VmWRKY13–VmWRKY25**	**87/102 (85.3%)**	**96/102 (94.1%)**	**0/102 (0.0%)**	**483**	**Y**
**VmWRKY62–VmWRKY55**	**98/164 (59.8%)**	**116/164 (70.7%)**	**14/164 (8.5%)**	**491**	**Y**
**VmWRKY2–VmWRKY16**	**378/571 (66.2%)**	**431/571 (75.5%)**	**20/571 (3.5%)**	**1865,5**	**Y**
**VmWRKY1–VmWRKY3**	**436/445 (98.0%)**	**437/445 (98.2%)**	**4/445 (0.9%)**	**2310,5**	**Y**
VmWRKY5–VmWRKY35	180/469 (38.4%)	238/469 (50.7%)	85/469 (18.1%)	686,5	N
**VmWRKY58–VmWRKY29**	**158/183 (86.3%)**	**166/183 (90.7%)**	**10/183 (5.5%)**	**803**	**Y**
VmWRKY68–VmWRKY50	96/402 (23.9%)	140/402 (34.8%)	121/402 (30.1%)	115,5	N
VmWRKY53–VmWRKY60	156/391 (39.9%)	206/391 (52.7%)	81/391 (20.7%)	615	N

Figure S4: Pairwise identities between paralogous pairs of *VmWRKY* genes - **Y**:yes, N: no
