# Supplementary material for: Genome-Wide Identification of Bilberry WRKY Transcription Factors: Go Wild and Duplicate
Source: Plants (Basel). 2023 Sep 5;12(18):3176. doi: 10.3390/plants12183176 (PMC10535657; doi:10.3390/plants12183176)
Supplement: Supplementary file 1 [file plants-12-03176-s001.zip › Figure S6.pdf]

Genome-Wide Expression of WRKY Transcription Factors in Blueberry: Go Wild and Duplicate

Winder Felipez [1,2,†], Jennifer Villavicencio [2,3,†], Valeria Oliveira Nizolli [2], Camila Pegoraro [2], Luciano da Maia [2] and Antonio Costa de Oliveira [2,*]

Duplicate Gene Pair	Ka	Ks	Ka_Ks	Duplicated Type	Time (Mya)*
VmWRKY37/VmWRKY38	NaN	NaN	NaN	Tandem	–
VmWRKY40/VmWRKY41	2.22107	2.59995	0.854276	Tandem	2.30431
VmWRKY46/VmWRKY47	NaN	NaN	NaN	Tandem	–
VmWRKY48/VmWRKY49	3.09421	2.55858	1.20934	Segmental	2.97652
VmWRKY4/VmWRKY19	NaN	NaN	NaN	Segmental	–
VmWRKY65/VmWRKY24	4.01533	NaN	NAN	Segmental	2.89474
VmWRKY11/VmWRKY44	NaN	NaN	NaN	Segmental	–
VmWRKY14/VmWRKY32	3.34641	4.13312	0.809656	Segmental	3.5265
VmWRKY61/VmWRKY63	3.66742	NaN	NaN	Segmental	2.59914
VmWRKY59/VmWRKY28	NaN	NaN	NaN	Segmental	–
VmWRKY67/VmWRKY22	2.95632	2.98684	0.989782	Segmental	2.96282
VmWRKY13/VmWRKY25	3.09497	NaN	NaN	Segmental	2.20679
VmWRKY62/VmWRKY55	4.39217	2.23753	1.96295	Segmental	3.91536
VmWRKY2/VmWRKY16	2.80634	NaN	NaN	Segmental	1.95985
VmWRKY1/VmWRKY3	NaN	NaN	NaN	Segmental	–
VmWRKY58/VmWRKY29	NaN	NaN	NaN	Segmental	–

**Mya, million years ago*

Figure S6: Ks, Ka, and Ks/Ks calculation and divergence time of duplicated bilberry *VmWRKY* gene pairs using using the KaKs_Calculator2.0 program, applying the Nei and Gojobori (NG) method the Nei and Gojobori (NG) method.

NaN = Not a Number (Refers to an undefined value or a result that cannot be calculated)
