# Supplementary material for: Genome-Wide Identification of Bilberry WRKY Transcription Factors: Go Wild and Duplicate
Source: Plants (Basel). 2023 Sep 5;12(18):3176. doi: 10.3390/plants12183176 (PMC10535657; doi:10.3390/plants12183176)
Supplement: Supplementary file 1 [file plants-12-03176-s001.zip › Figure S7.pdf]

Genome-Wide Expression of WRKY Transcription Factors in Blueberry: Go Wild and Duplicate

Winder Felipez [1,2,†], Jennifer Villavicencio [2,3,†], Valeria Oliveira Nizolli [2], Camila Pegoraro [2], Luciano da Maia [2] and Antonio Costa de Oliveira [2,*]

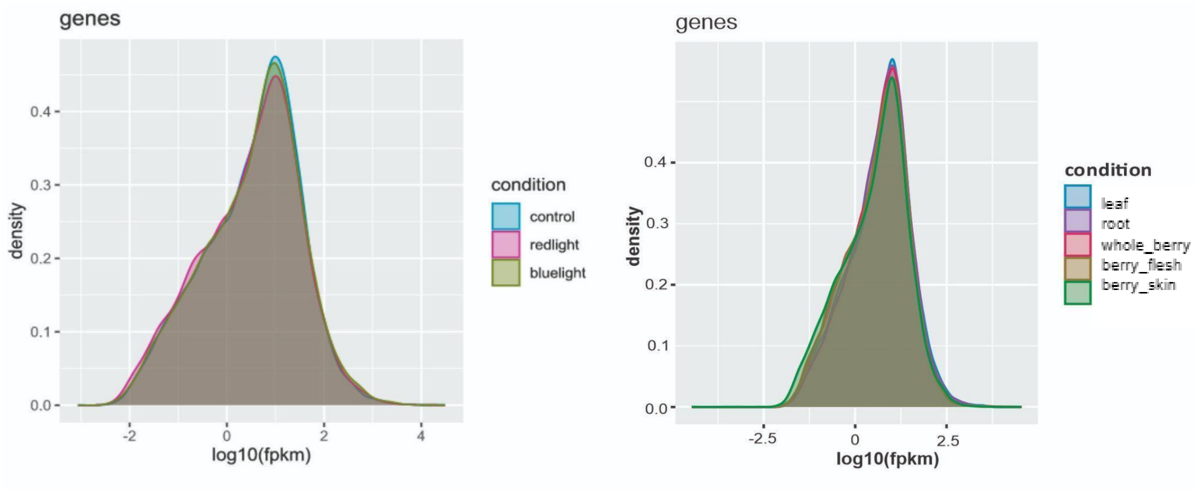

Figure S7: Analysis of gene expression in samples of *Vaccinium myrtillus*: A) CummeRbund plots of the expression level distribution for all genes in simulated experimental conditions control, red light and blue light. FPKM, fragments per kilobase of transcript per million fragments mapped, B) CummeRbund plots of the expression level distribution for all genes in leaf, root, whole berry, berry flesh and berry skin. FPKM, fragments per kilobase of transcript per million fragments mapped.
