# Supplementary material for: Genome-Wide Identification of Bilberry WRKY Transcription Factors: Go Wild and Duplicate
Source: Plants (Basel). 2023 Sep 5;12(18):3176. doi: 10.3390/plants12183176 (PMC10535657; doi:10.3390/plants12183176)
Supplement: Supplementary file 1 [file plants-12-03176-s001.zip › Figure S8.pdf]

Genome-Wide Expression of WRKY Transcription Factors in Blueberry: Go Wild and Duplicate

Winder Felipez [1,2,†], Jennifer Villavicencio [2,3,†], Valeria Oliveira Nizolli [2], Camila Pegoraro [2], Luciano da Maia [2] and Antonio Costa de Oliveira [2,*]

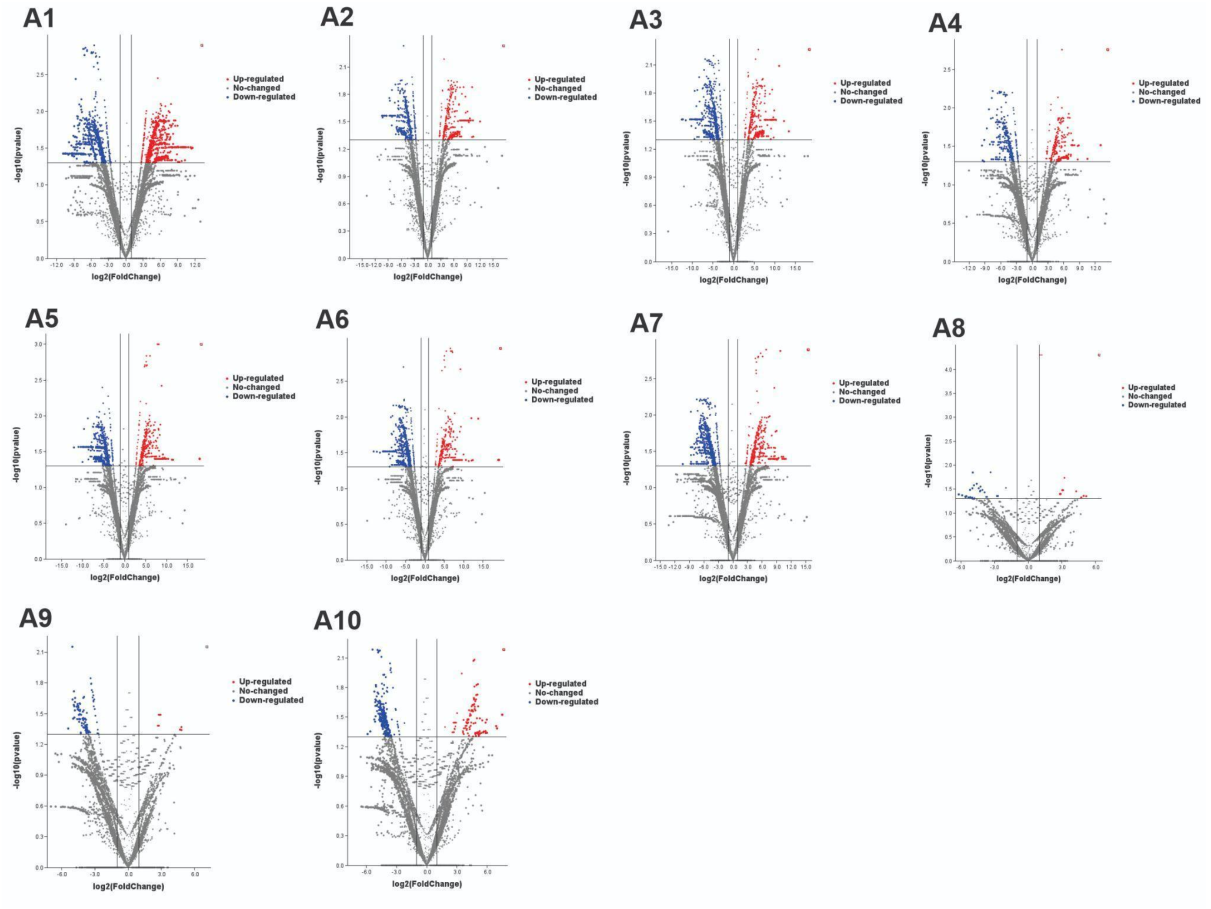

Figure S8: Analysis of gene expression in samples of *Vaccinium myrtillus*: A1 - A10) Volcano plots reveal genes, that differ significantly between the pairs leaf vs root, Leaf vs whole berry, leaf vs berry flesh, leaf vs berry skin, root vs whole berry, root vs berry flesh, root vs berry skin, whole berry vs berry flesh, whole berry vs berry skin and berry flesh vs berry skin.
