# Supplementary material for: Genome-Wide Identification of Bilberry WRKY Transcription Factors: Go Wild and Duplicate
Source: Plants (Basel). 2023 Sep 5;12(18):3176. doi: 10.3390/plants12183176 (PMC10535657; doi:10.3390/plants12183176)
Supplement: Supplementary file 1 [file plants-12-03176-s001.zip › Figure S9.pdf]

Genome-Wide Expression of WRKY Transcription Factors in Blueberry: Go Wild and Duplicate

Winder Felipez [1,2,†], Jennifer Villavicencio [2,3,†], Valeria Oliveira Nizolli [2], Camila Pegoraro [2], Luciano da Maia [2] and Antonio Costa de Oliveira [2,*]

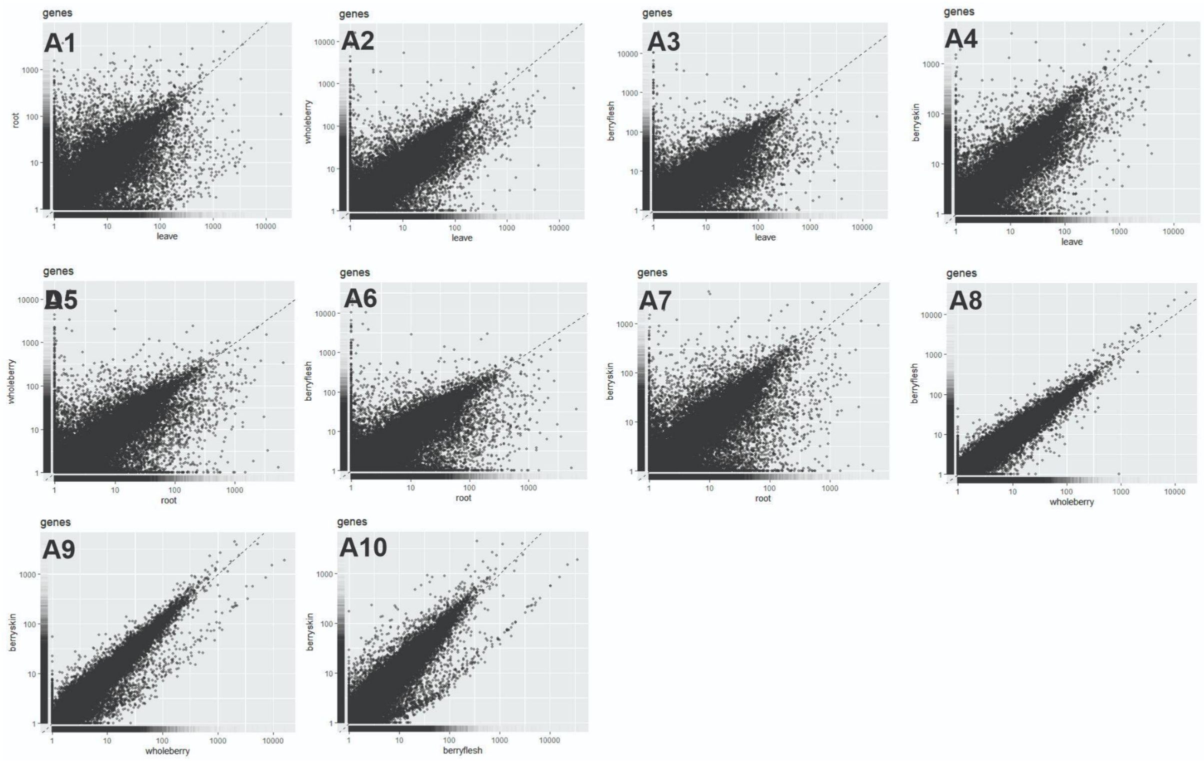

Figure S9: Analysis of gene expression in samples of *Vaccinium myrtillus*: D1 - D10) CummeRbund scatter plots highlight general similarities and specific outliers between conditions root and leaf, whole berry and leaf, berry flesh and leaf, berry skin and leaf, whole berry and root, berry flesh and root, berry skin and root, berry flesh and whole berry, berry skin and whole berry, berry skin and berry flesh.
