# Supplementary material for: Genome-Wide Identification of Bilberry WRKY Transcription Factors: Go Wild and Duplicate
Source: Plants (Basel). 2023 Sep 5;12(18):3176. doi: 10.3390/plants12183176 (PMC10535657; doi:10.3390/plants12183176)
Supplement: Supplementary file 1 [file plants-12-03176-s001.zip › Figure S1.pdf]

Genome-Wide Expression of WRKY Transcription Factors in Blueberry: Go Wild and Duplicate

Winder Felipez [1,2,†], Jennifer Villavicencio [2,3,†], Valeria Oliveira Nizolli [2], Camila Pegoraro [2], Luciano da Maia [2] and Antonio Costa de Oliveira [2,*]

Figure S1: Schematic representation of conserved motifs identified in bilberry VmWRKY proteins.

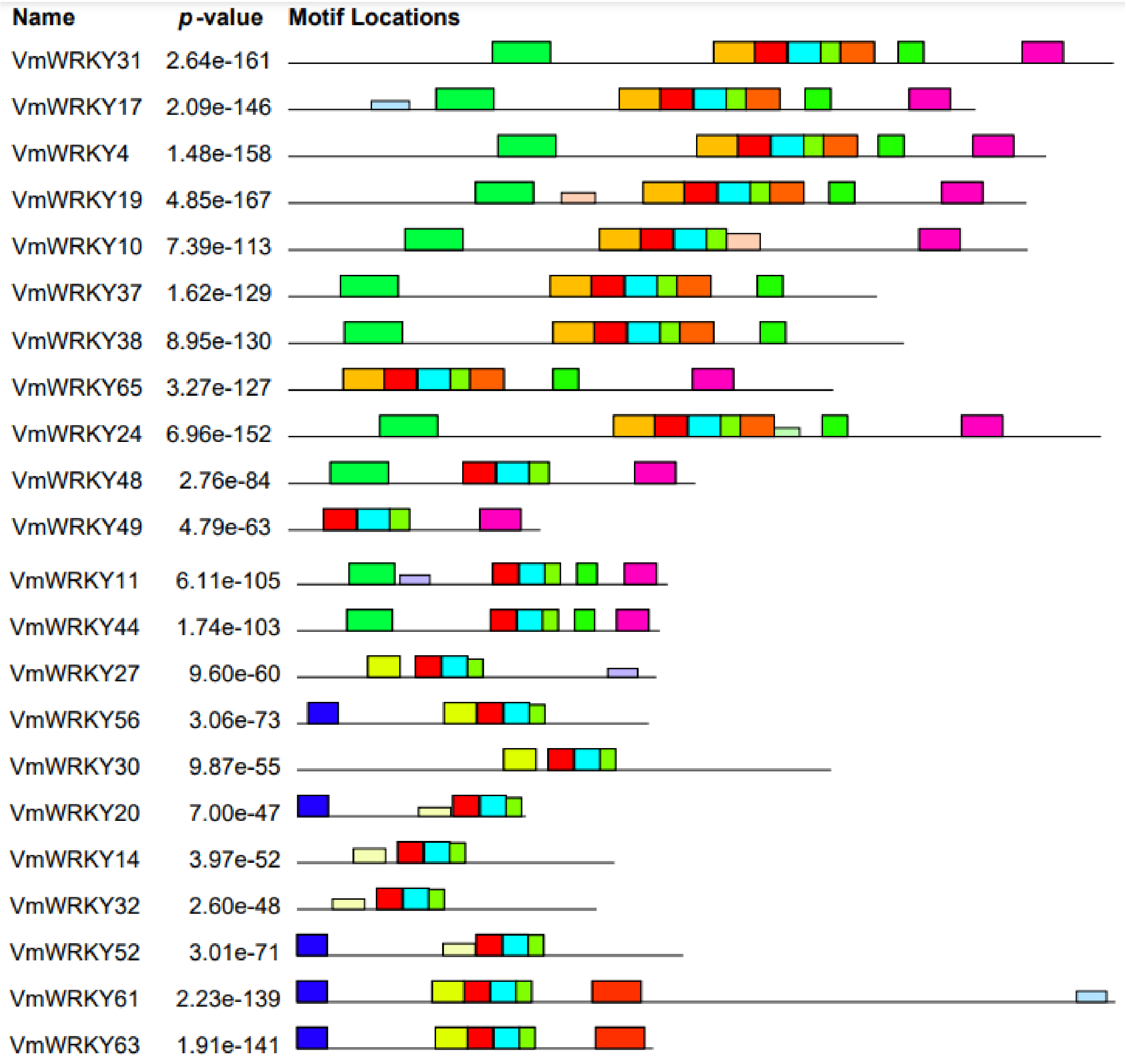

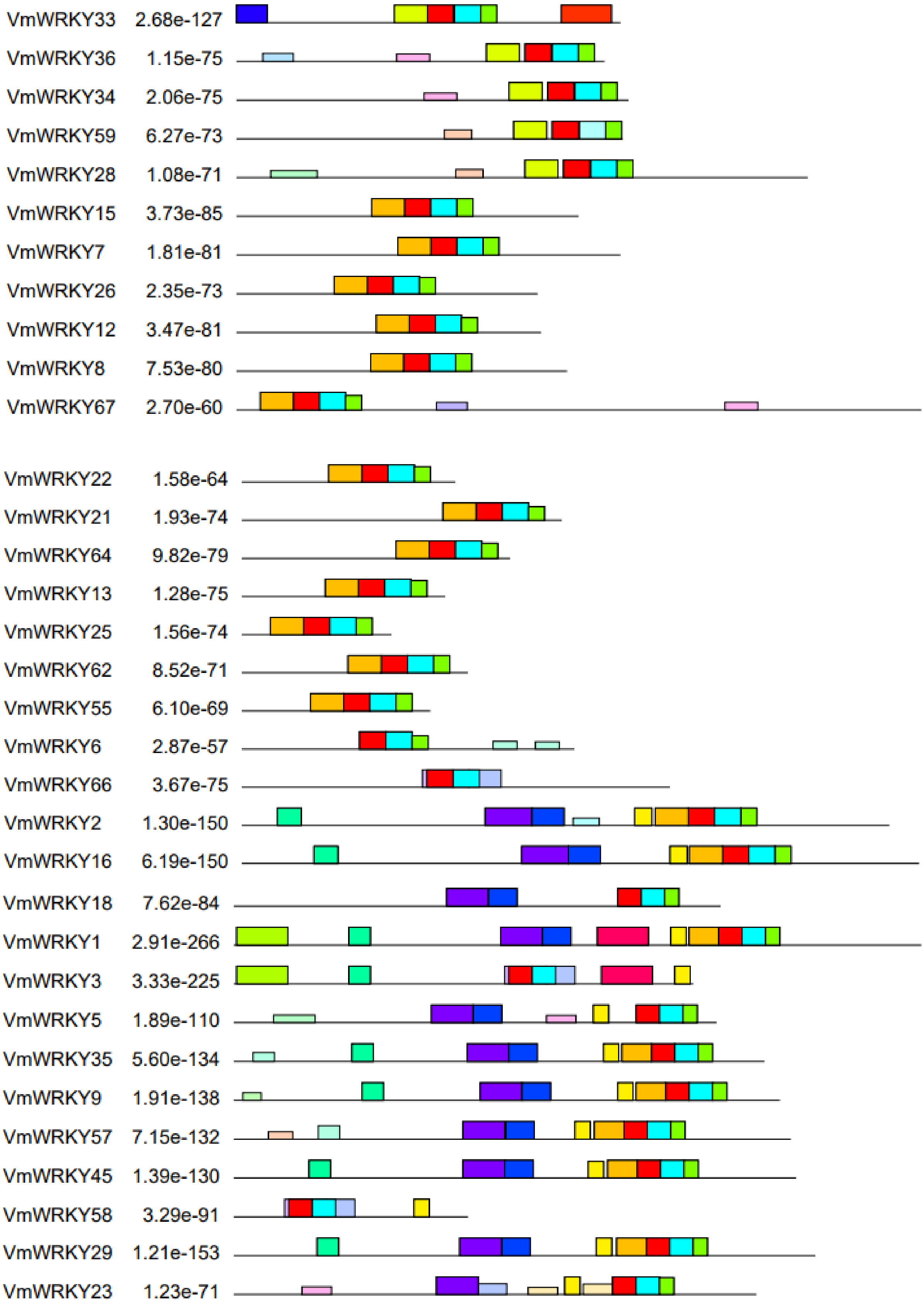
VmWRKY33 2.68e-127
VmWRKY36 1.15e-75
VmWRKY34 2.06e-75
VmWRKY59 6.27e-73
VmWRKY28 1.08e-71
VmWRKY15 3.73e-85
VmWRKY7 1.81e-81
VmWRKY26 2.35e-73
VmWRKY12 3.47e-81
VmWRKY8 7.53e-80
VmWRKY67 2.70e-60
VmWRKY22 1.58e-64
VmWRKY21 1.93e-74
VmWRKY64 9.82e-79
VmWRKY13 1.28e-75
VmWRKY25 1.56e-74
VmWRKY62 8.52e-71
VmWRKY55 6.10e-69
VmWRKY6 2.87e-57
VmWRKY66 3.67e-75
VmWRKY2 1.30e-150
VmWRKY16 6.19e-150
VmWRKY18 7.62e-84
VmWRKY1 2.91e-266
VmWRKY3 3.33e-225
VmWRKY5 1.89e-110
VmWRKY35 5.60e-134
VmWRKY9 1.91e-138
VmWRKY57 7.15e-132
VmWRKY45 1.39e-130
VmWRKY58 3.29e-91
VmWRKY29 1.21e-153
VmWRKY23 1.23e-71

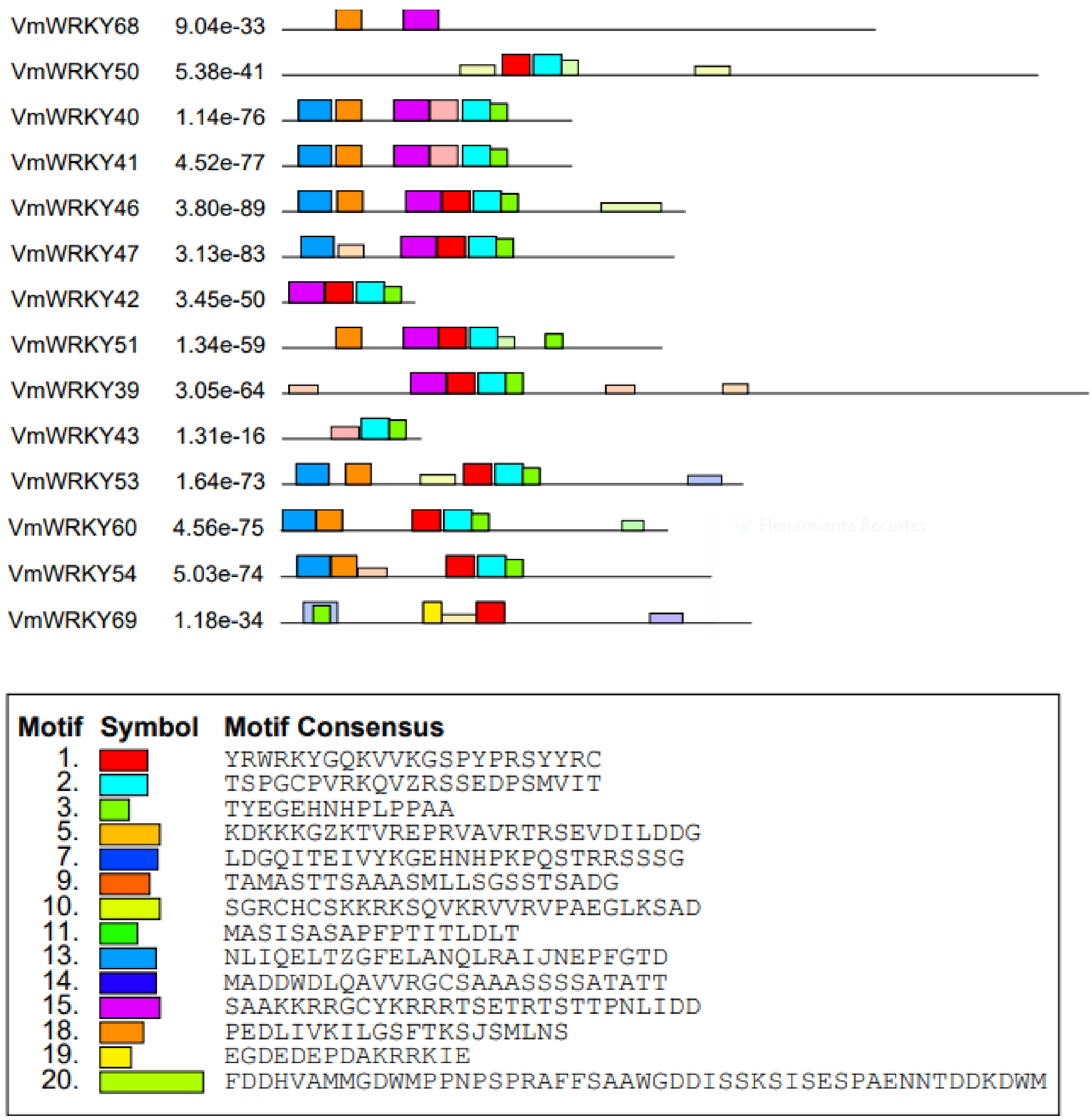
VmWRKY68 9.04e-33
VmWRKY50 5.38e-41
VmWRKY40 1.14e-76
VmWRKY41 4.52e-77
VmWRKY46 3.80e-89
VmWRKY47 3.13e-83
VmWRKY42 3.45e-50
VmWRKY51 1.34e-59
VmWRKY39 3.05e-64
VmWRKY43 1.31e-16
VmWRKY53 1.64e-73
VmWRKY60 4.56e-75
VmWRKY54 5.03e-74
VmWRKY69 1.18e-34
Motif Symbol Motif Consensus
1. YRWRKYGQKVVKGSPYPRSYYRC
2. TSPGCPVRKQVZRSSEDPSMVIT
3. TYEGEHNHPLPPAA
5. KDKKKGZKTVREPRVAVRTRSEVDILDDG
7. LDGQITEIVYKGEHNHPKPQSTRRSSSG
9. TAMASTTSAAASMLLSGSSTSADG
10. SGRCHCSKKRKSQVKRVVRVPAEGLKSAD
11. MASISASAPFPTITLDLT
13. NLIQELTZGFELANQLRAIJNEPFGTD
14. MADDWDLQAVVRGCSAAASSSSATATT
15. SAAKKRRGCYKRRRTSETRTSTTPNLIDD
18. PEDLIVKILGSFTKSJSMLNS
19. EGDEDEPDAKRRKIE
20. FDDHVAMMGDWMPPNPSPRAFFSAAWGDDISSKSISESPAENNTDDKDWM
